# Editorial: Clinical management, pathogenesis and biomarkers of cardiovascular disease associated with systemic autoimmune disorders

**DOI:** 10.3389/fimmu.2022.1085875

**Published:** 2022-11-23

**Authors:** Carlos Perez-Sanchez, Inés Pineda-Torra, Chary Lopez-Pedrera

**Affiliations:** ^1^ Maimonides Biomedical Research Institute of Cordoba (IMIBIC), Cordoba, Spain; ^2^ University of Cordoba, Cordoba, Spain; ^3^ Hospital Universitario Reina Sofía, Cordoba, Spain; ^4^ Andalusian Center of Molecular Biology and Regenerative Medicine, Spanish National Research Council (CSIC), Seville, Spain

**Keywords:** cardiovascular disease, autoimmune disease, inflammatory disease, biomarker, pathogenesis

In this Research Topic several studies have analysed the clinical and molecular profile related to cardiovascular disease (CVD) development in a range of inflammatory and systemic autoimmune diseases, including systemic lupus erythematosus (SLE), psoriatic arthritis, rheumatoid arthritis and spondylarthritis among others. Novel molecular mechanisms and biomarkers have been characterized along with clinical tools and approaches to manage this prevalent comorbidity.

In the case of SLE, three studies have explored at the molecular level the underlaying mechanisms associated with CVD. Quevedo-Abeledo et al., studied the levels of three CVD molecules involved in the metabolism of triglycerides, such as lipoprotein lipase (LPL), angiopoietin-like protein 4 (ANGPLT4), and apolipoprotein C-III (ApoC3), in 185 patients with SLE and 162 healthy donors. Performing a multivariable analysis, they identified that the LPL, ANGPLT4 and ApoC3, axis is altered in SLE and associated with the disease damage score. Wang et al., used public transcriptomic data to gain insight into the involvement of new players and pathways related to the development of atherosclerosis in SLE patients. They identified 5 hub genes (SIGLEC1, CD163, CCR1, MMP9, and IL1RN) which might promote the monocytes differentiation into macrophages and the pathway of IL-17 signalling as potential mechanisms involved in the atherosclerotic process of SLE patients. Guzman-Martinez et al., reviewed the association of the immune system with the pathogenesis of endothelial damage and atherosclerosis in SLE patients, including inflammatory mediators, specific circulating cell populations and autoantibodies. Additionally, they discussed the utility of the immune system as early CVD biomarkers and therapeutic targets in SLE.

On the other hand, Remuzgo-Martínez et al., analysed the role of the protein irisin as a serological and genetic biomarker of CV risk, disease severity and subclinical atherosclerosis in a cohort of 725 patients with axial spondylarthritis (axSpA). Their results suggested that low levels of irisin in the serum could be considered a marker of high CV risk, more severe disease and the presence of subclinical atherosclerosis, in axSpA patients. Furthermore, they found that irisin may also constitute a biomarker of disease activity in axSpA at the genetic level.

In the case of inflammatory arthritis like rheumatoid arthritis (RA) and psoriatic arthritis (PsA), Barbarroja et al., reviewed the interplay of hepatic alterations and CVD, analysing different mechanisms where autoimmunity, chronic inflammation, metabolic deregulation and treatments seem to have an key role. They also discussed the latest controversies regarding the effects of anti-inflammatory therapies used in PsA and RA in the liver damage, such as biologics and DMARDs such as, leflunomide or methotrexate. Schwartz et al., also analyzed in a cohort of PsA patients, the associations of metabolic dysregulation and systemic inflammation with coronary artery disease (CAD) measuring traditional CVD risk factors, serum markers of metabolic dysfunction, inflammatory cytokines and inflammation in specific tissues by using positron emission tomography-computed tomography (PET-CT). They identified metabolic and inflammatory players associated with subclinical CAD in PsA, including inflammation in adipose tissue which might be considered as novel target for CVD prevention and treatment in PsA.

The association between Psoriasis and the risk of CVD was also explored by Gao et al., using mendelian randomization and genome-wide association study (GWAS). The analysis suggested a potential causal link between CVD and psoriasis.

Lastly, two studies in this issue evaluated the CV involvement in rare systemic autoimmune disorders.

Zhou et al., analyzed in anti-melanoma differentiation-associated gene 5 (anti-MDA5) antibody-positive dermatomyositis (DM)//clinically amyopathic dermatomyositis (anti-MDA5 Ab+ DM/CADM) the myocardial involvement. Anti-MDA5 dermatomyositis is a rare autoimmune disease, with high prevalence in Japanese patients with clinically amyopathic dermatomyositis and progressive interstitial lung disease. It also shows systemic manifestations affecting skeletal muscle, skin, and other organs.

In a cohort of seventy-six hospitalized patients suffering this disorder, this study identified twelve patients with MI (16%), associated with severe cardiovascular complications and adverse evolution of the disease. They concluded that myocardial involvement is an independent poor prognostic factor of patients with anti-MDA5 Ab+ DM/CADM.

Huang et al., investigated the efficacy of percutaneous transluminal pulmonary angioplasty (PTPA) in patients with Takayasu arteritis (TA) and pulmonary hypertension (PH) and pulmonary artery stenosis.

Takayasu’s arteritis is a rare chronic inflammatory autoimmune condition that impact the largest blood vessels in the body (aorta) and its branches, the supra-aortic trunks.

Results of this study, analyzing 183 lesions from 79 surgeries carried out on 32 patients with TA and PH, indicated that PTPA improved clinical symptoms, hemodynamic parameters, and exercise tolerance, in patients with TA pulmonary artery stenosis and PH. Furthermore, reperfusion pulmonary edema was significantly reduced, and no patient died of PTPA-related complications with guidance from the pressure wire.

Overall, these findings might be important for healthcare resource planning and preventive approaches. These 9 articles substantial CV-risk observed in autoimmune diseases patients, suggests that strategies to reduce CV-risk should become a routine part of the management of autoimmune disease patients. However, the causes of cardiovascular involvement associated with systemic autoimmune diseases, and their potential therapy, require further research.

## Author contributions

All authors listed have made a substantial, direct, and intellectual contribution to the work and approved it for publication.

## Conflict of interest

The authors declare that the research was conducted in the absence of any commercial or financial relationships that could be construed as a potential conflict of interest.

## Publisher’s note

All claims expressed in this article are solely those of the authors and do not necessarily represent those of their affiliated organizations, or those of the publisher, the editors and the reviewers. Any product that may be evaluated in this article, or claim that may be made by its manufacturer, is not guaranteed or endorsed by the publisher.

